# Emissions generated by sugarcane burning promote genotoxicity in rural workers: a case study in Barretos, Brazil

**DOI:** 10.1186/1476-069X-12-87

**Published:** 2013-10-10

**Authors:** Henrique César Santejo Silveira, Marina Schmidt-Carrijo, Ervald Henrique Seidel, Cristovam Scapulatempo-Neto, Adhemar Longatto-Filho, Andre Lopes Carvalho, Rui Manuel Vieira Reis, Paulo Hilário Nascimento Saldiva

**Affiliations:** 1 Molecular Oncology Research Center, Barretos Cancer Hospital, Barretos, SP, Brazil; 2 Life and Health sciences Research Institute (ICVS), University of Minho, Braga, Portugal; 3 ICVS/3B’s-PT Government Associate Laboratory, Braga/Guimarães, Portugal; 4 Laboratory of Medical Investigation (LIM) 14, Faculty of Medicine, University of São Paulo, São Paulo, Brazil; 5 Department of Pathology, Faculty of Medicine, University of São Paulo, São Paulo, Brazil; 6 Department of Pathology, Barretos Cancer Hospital, Barretos, SP, Brazil

**Keywords:** Sugar cane workers, Micronuclei, Genomic instability, Human lymphocytes, Exfoliated buccal cells

## Abstract

**Background:**

To determine the possible genotoxic effect of exposure to the smoke generated by biomass burning on workers involved in manual sugar cane harvesting.

**Methods:**

The frequency of micronuclei in exfoliated buccal cells and peripheral blood lymphocytes was determined in sugarcane workers in the Barretos region of Brazil, during the harvest season and compared to a control population, comprised of administrative employees of Barretos Cancer Hospital.

**Results:**

The frequency of micronuclei was higher in the sugar cane workers. The mean frequency in blood lymphocytes (micronuclei/1000 cells) in the test group was 8.22 versus 1.27 in the control group. The same effect was observed when exfoliated buccal cells were considered (22.75 and 9.70 micronuclei/1000 cells for sugar cane workers and controls, respectively).

**Conclusion:**

Exposure to emissions produced by the burning of sugar cane during harvesting induces genomic instability in workers, indicating the necessity of adopting more advanced techniques of harvesting sugar cane to preserve human health.

## Background

Biofuels have been considered a cleaner and more sustainable alternative compared to fossil fuels. To reduce the emissions of greenhouse gases as well as local pollutants, several countries are including biofuels in their energy policies. Sugar cane ethanol is one of the most widely used sources of ethanol in Brazil, which has run a program for using ethanol as an automotive fuel since the mid-1970s. Thus, Brazil contains 25% of the total land worldwide that is planted with sugarcane. The state of São Paulo is the largest producer of this crop; between 2001 and 2011, the production of sugarcane grew 121% due to the use of biofuels in cars.

When projecting a future scenario with an increased production, it is important to focus on the health effects associated with the different steps of sugar cane production. Currently, the sugarcane harvest is associated with straw burning for reasons of productivity as well as to avoid contact of workers with the sharp leaves and poisonous animals in the sugar cane plantation. However, the burning process results in a high exposure to smoke, which is still present during harvest.

The environmental impact due to the burning of sugar cane has diminished with the implementation of legislation to suspend the use of burning to use mechanized harvesting as an alternative in São Paulo [1]. These laws have not been extended to the rest of Brazil, where sugarcane plantations are expanding, and in the Central America and Africa, where the burning of sugar cane is still practiced [2].

Biomass burning is a major source of toxic gases [3]. Several products are generated during this process that result in adverse effects on the health of the exposed population [4], such as particulate matter, polycyclic aromatic hydrocarbons (PAHs), carbon monoxide, aldehydes, organic acids, volatile and semi-volatile compounds of nitrogen and sulfur, ozone and inorganic chemical species. Taking into account the health effects associated with sugar cane burning, it is important to focus on fine and ultrafine particulate matter (PM10 and PM2.5), which consists of a mixture of liquids, gases and solids deposited on particles such as PAHs, which are derived from an incomplete organic combustion process [5]. PAHs are pollutants that cause mutagenic and carcinogenic effects [6,7]. In the Brazilian city of Araraquara (an area with high production of sugar cane in São Paulo State), a significant increase in PAHs, especially the benzopyrene fractions of particulate matter (PM10 and PM2.5), occurs during the sugar cane harvest [7]. Populations in areas surrounded by sugarcane plantations are exposed to the particles produced by biomass burning continuously for at least six months to a year, and increased hospital admissions due to asthma occur during these periods [8,9].

Sugar cane workers are exposed to high levels of particulate matter, thermal overload, and intense physical exertion during the harvest period, and these conditions induce muscle lesions, changes in blood coagulation and heart rate, systemic oxidative stress, and high blood pressure [10]. Sugarcane products have been shown to cause respiratory problems in workers [8]. Furthermore, sugar cane workers are exposed to various genotoxic compounds, including PAHs [11]. Considering the aforementioned evidence, it is important to evaluate genomic instability in sugarcane workers. Micronucleus (MN) assessment is a biomarker test of genotoxic events and manifestations of chromosomal instability that are frequently observed in diseases such as cancer and can thus evaluate the potential risk of disease in exposed populations [12]. The MN test in blood lymphocytes and buccal mucosal cells is widely used to assess the extension of chromosomal genetic alterations promoted by the exposure to environmental toxicants [13-15]. The aim of the present study was to evaluate the MN frequencies in blood lymphocytes and exfoliated buccal cells of sugar cane workers during the harvest season.

## Methods

### Study groups

The present study was approved by the Barretos Cancer Hospital ethical committee (n˚ 361/2010), and all participants signed an informed consent prior to any intervention.

The study population consisted of a group of male sugarcane workers (n = 23) in the Barretos region, and the control group (n = 30) was taken from the administrative staff of Barretos Cancer Hospital. The age of the subjects under study ranged from 22 to 45 years. Every participant answered a questionnaire including general information and occupational data, such as previous occupational background, working time in the current job and past and previous smoking habits.

### Determination of micronucleus frequency in blood lymphocytes

Blood was collected in vacuum tubes (BD TriPath Imaging, Burlington, N.C., USA). The samples were transported carefully to avoid heat and agitation. Blood lymphocytes were isolated with FicollPaque (Invitrogen). The cultures were grown in RPMI medium at 37°C for 72 hours, and phytohemagglutinin was added (Lifetechnologies). After 44 hours, citochalasin B (Sigma) was added to a final concentration of 6 μg/mL following [16]. Then, to resuspend the cells after cultivation for 72 hours, SurePath preservative liquid (BD TriPath Imaging, Burlington, N.C., USA) was used with Liquid-based cytology medium (BD TriPath Imaging, Burlington, N.C., USA), to ensure a single layer of cells with excellent smear quality. This fixative solution is a mixture of ethanol, methanol and isopropyl alcohol. One ml of this solution was transferred to an incubation chamber mounted on a support (BD TriPath Imaging, Burlington, N.C., USA) consisting of a PrepStain Settling Chamber (BD TriPath Imaging, Burlington, N.C., USA) placed on the top of a glass slide. A cell suspension from each sample was dropped onto a clean glass slide. After one hour, the slides were stained automatically, as specified by the manufacturer. The slides were previously encoded and analyzed under an optical microscope at a magnification of 1000X. One thousand cells were counted per sample. The MN frequencies were expressed as MN per thousand cells. All experiments were carried out in duplicate.

### Determination of micronucleus frequency in exfoliated buccal cells

Exfoliated buccal cells were sampled with the assistance of a cytobrush (BD TriPath Imaging, Burlington, N.C., USA), which was stirred in Surepath preservative liquid (BD TriPath Imaging, Burlington, N.C., USA). The material was centrifuged at 1500 rpm for 10 minutes, and the supernatant was discarded, thus obtaining a precipitate with a high concentration of cells. Smears were produced and stained with Papanicolaou staining. Then, the cells were dehydrated in ethanol and clarified in xylene. The slides were mounted with coverslips. Then, 1000 cells were counted, and the micronucleus frequencies in both groups were determined. The slides were stained by Papanicolaou staining, 1000 cells per sample were counted, and the MN frequencies were determined. The cells were counted under an optical microscope using a magnification of 1000X.

### Statistical analysis

Statistical analysis was conducted with the aid of SPSS software for Windows version 19.0. Initially, the data were tabulated based on descriptive statistics (mean, standard deviation, minimum, maximum and quartiles) for quantitative data and frequency tables for qualitative data. The mean MN frequencies in lymphocytes and exfoliated buccal cells in the control group and the sugarcane workers’ group were compared with Student’s t-test, for which the samples were considered independent groups. A p-value < 0.05 was considered statistically significant in all analyses.

## Results

The characteristics of the groups are presented in Table 1. The age and smoking habits were similar in the exposed and control groups. Only one individual from each group had a history of occupation-related disease. Four sugar cane workers and three controls were using medications that are not associated with genotoxic effects (Table 1).

**Table 1:** Demographic characteristics of the subjects

	**Controls**	**Sugarcane cutters**
Individuals (N)	30	23
Mean age (± SD*)	30.50 (5.5)	31.00 ( 6.7)
Work hours, mean (± SD)	46.33 (8.3)	51.39 (3.9)
N** of Smokers (± SD)	7	5
N** Ex-smokers	1	0
Mean age for beginning cigarette smoking (± DP)	17.00 (2.0)	16.40 (2.3)
N Risk of inhalation of harmful substances*** (in earlier jobs)	9	-
N Medication (Current)	30	21
No (%)	26 (86.7)	18 (85.8)
Yes (%)****	4 (13.3)	3 (14.2)
N Diseases related to work	30	23
Yes (%)*****	1 (3.3)	1 (4.3)
No (%)	29 (96.7)	22 (95.7)

SD* = Standard Deviation; N** = Number of subjects; ***Harmful substances: dust, smoke, gases and chemicals; ****Anti-inflammatory, anti-allergic, anti-muscarinic, antihypertensive and analgesic; *****1 control with wrist synovial tendinitis and 1 sugar cane cutter with muscle aches and cramps.

(−) Not applied to the cutter group.

A total of 51,000 binucleated lymphocytes were analyzed for the verification of micronuclei. The MN frequencies in peripheral lymphocytes (micronuclei/1000 cells and Additional file 1) were higher (p < 0.001) in the sugar cane workers (mean = 8.22, SD = 4.18) compared with the control group (mean = 1.27, SD = 1.34), as shown in Table 2.

**Table 2:** Comparison of the MN frequencies by Student’s t-test, considering independent groups

					**Confidence interval**		
		**Mean**	**SD**	**DM**	**Lower**	**Upper**	***p-value***
Lymphocytes	Control (N = 30)	1.27	1.34	−6.95	−8.58	−5.32	<0.001
	Cutters (N = 23)	8.22	4.18				
Buccal cells	Control (N = 27)	9.70	4.76	−13.05	−16.33	−9.76	<0.001
	Cutters (N = 16)	22.75	5.78				

*N* number of subjects per group considered in the analysis, *SD* Standard deviation, *DM* differences of the mean, considered statistically significant when p <0.05.

**Figure 1:**
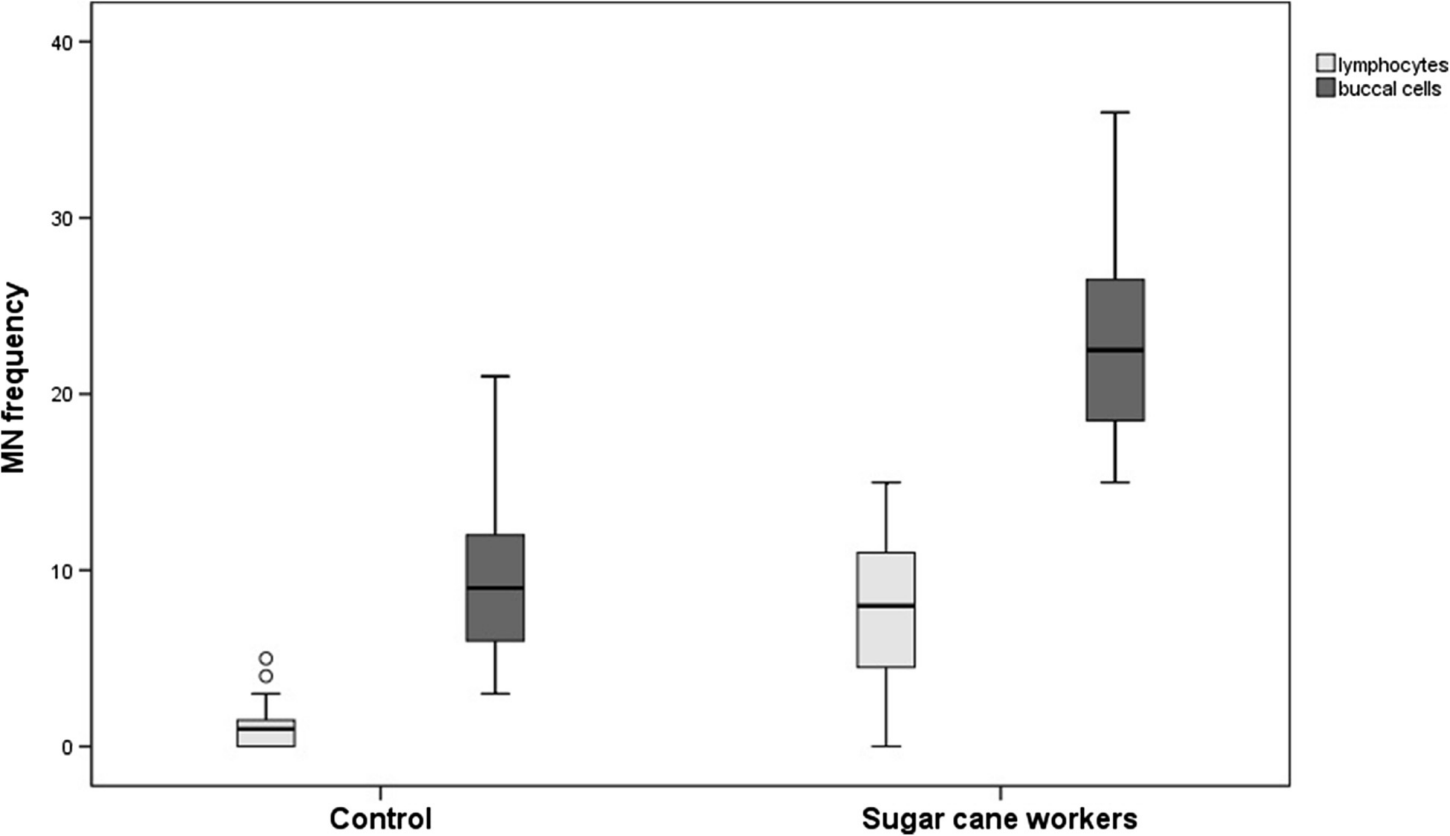
**Graphic representation of distribution of both groups according to their MN frequency.** The MN frequency in lymphocytes (light gray) and the MN frequencies of exfoliated buccal cells (dark gray) were considered for this analysis. (°) indicates discrepancy points, bars indicate percentile values, and lines indicate minimum and maximum standard deviations.

Adequate smears of exfoliated buccal cells were possible in 43 individuals (16 sugar cane workers and 27 controls) Additional file 1. Again, a higher micronucleus frequency in exfoliated cells was obtained in the group of sugar cane cutters (mean = 22.75, SD = 5.78) compared with the controls (mean = 9.70, SD = 4.76), with a statistically significant p-value <0.001 (Table 2). The distribution of the groups according to their MN frequencies is shown in Figure 1.

The MN frequency in exfoliated buccal cells was higher in comparison to that determined in pair-wise peripheral blood lymphocytes (the mean difference in the control and cutter groups was −8.59 and −13.87, respectively, p-value <0.001), as shown in Table 3.

**Table 3:** Analysis of mean MN frequencies versus MN frequencies of lymphocytes and MN frequencies of buccal cells

						**Confidence interval**		
		**Mean**	**SD**	**DM**	**SD of difference**	**Lower**	**Upper**	***p-value***
Control	Lymphocytes (N = 27)	1.11	1.28	−8.59	0.89	−10.43	−6.75	<0.001
	Buccal cells (N = 27)	9.70	4.76					
Cutters	Lymphocytes (N = 16)	8.87	4.05	−13.87	1.78	−17.68	−10.07	<0.001
	Buccal cells (N = 16)	22.75	5.78					

*N* number of subjects per group considered in the analysis, *SD* Standard Deviation, *DM* Differences of the mean, considered statistically significant when p < 0.05. We only considered both individuals for which the obtained information was collected in these analyses.

**Figure 2:**
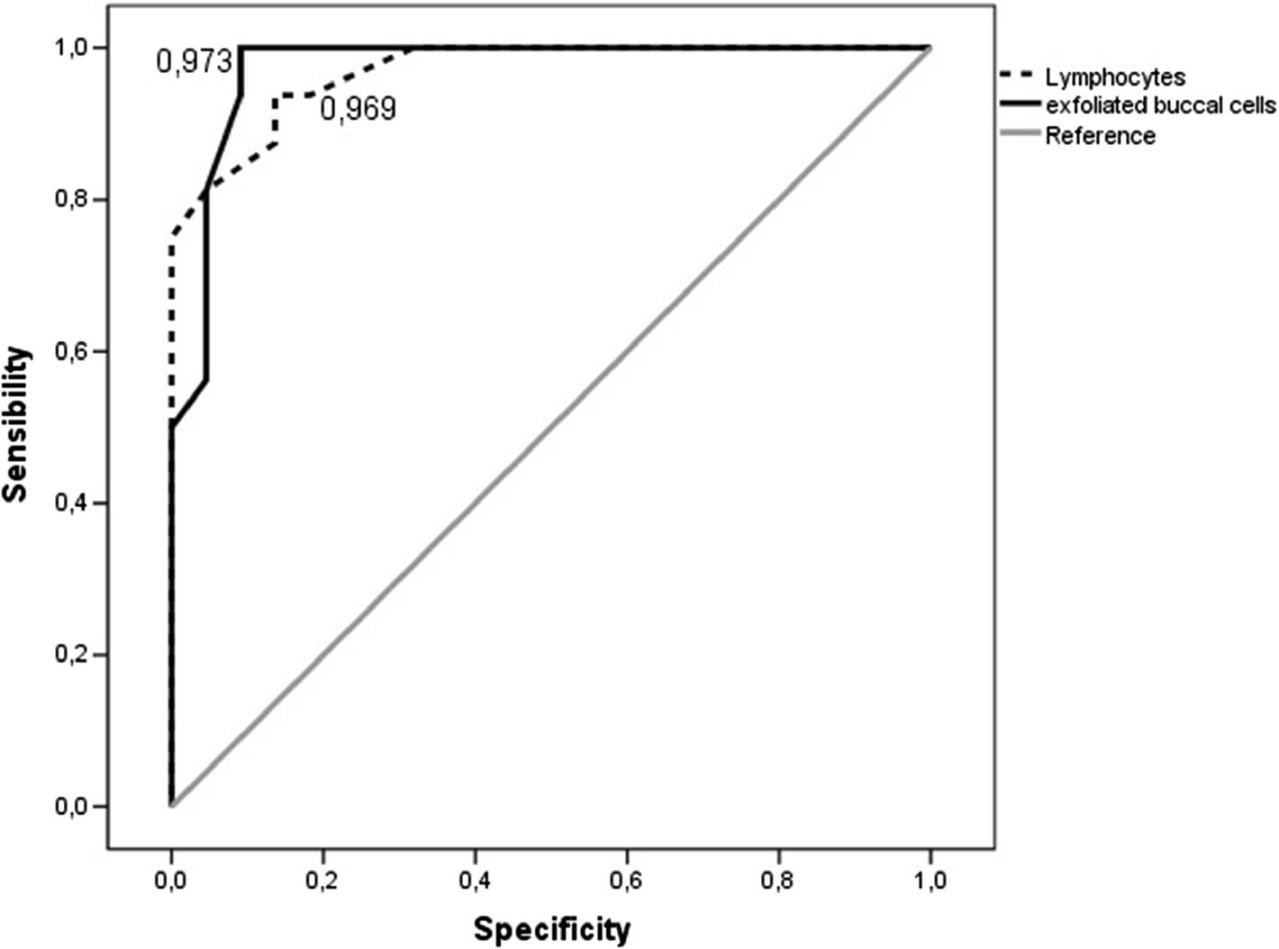
**Graph representing of the ROC curve.** Receiver operating characteristics curves of the MN frequency in lymphocytes (trace) and the MN frequency in cells exfoliated from the mouth (black). Reference line (gray). The proximity of curves to the upper left corner of the most accurate methods was analyzed.

Additionally, the Curve Receiver Operating Characteristics (ROC) curves were generated to evaluate the sensitivity and specificity of the two methods (peripheral lymphocytes and exfoliated mucosal cells) in the characterization of the controls and sugar cane workers. Thus, the respective areas of the ROC curves were 0.969 for the MN frequency in lymphocytes and 0.973 for the MN frequencies in exfoliated buccal cells (Figure 2).

## Discussion

The results obtained in the present study revealed that workers exposed to emissions generated by sugar cane burning exhibit a higher micronucleus frequency in peripheral blood lymphocytes and buccal mucosa exfoliated cells. Indeed, because the harvesting process begins when smoke emissions are still present, these workers are exposed to a high level of air pollution during intense physical activity (due to the intense effort required for manual cutting and harvesting), which demands higher pulmonary ventilation [10]. Considering the two biomarkers used in our study, our results indicate that this group of workers developed a significant degree of genotoxicity as a result of compounds emitted by biomass burning. The results are in agreement with a previous study by our group in the Brazilian Amazon region, in which MN frequencies in exfoliated buccal cells of children were utilized to assess the genotoxicity potential of biomass burning pollutants [17].

In recent decades, biomarkers have been used to assess exposure to genotoxic agents, and the increase in these biomarkers, such as MN, in early cellular events is associated with changes related to diseases such as cancer. Assuming that the cellular mechanisms for the induction of chromosomal damage are similar in different tissues, the extension of DNA damage in lymphocytes and other tissues most likely reflects the level of cancer risk [12,18]. Furthermore, studies have shown that in Western countries, lung cancer is a major cause of mortality related to malignant tumors, and its pathogenesis involves the accumulation of various molecular abnormalities during long periods of time and has been associated with exposure to PAHs [19-21].

Recently, a comparison of different studies that assessed the MN frequency in blood lymphocytes and exfoliated buccal cells of the same individuals simultaneously revealed a strong correlation between the MN frequencies in both tissues [22]. Thus, it is possible that measurements of MN frequency in oral epithelial cells can be potentially used as a screening alternative for larger studies focusing on cancer risk [23,24]. Thus, MN scoring can be used as a biomarker to identify several pre-neoplastic conditions much earlier than the clinical manifestations appear [25]. MN scoring in occupational groups reporting exposure to solvents, polycyclic aromatic hydrocarbons, gasoline, arsenic and antineoplastic drugs showed an increase in the MN frequency compared to the corresponding control group [12]. An increase in MN frequency is suggestive of but not diagnostic for cancer risk. However, MN frequency should be considered with respect to the detailed clinical history and examination [25] because MN frequency is the result of the balance between exposure to genotoxic agents and the genetic susceptibility of each individual [25]. Interestingly, previous studies have reported associations of MN frequency in peripheral blood lymphocytes not only with cancer risk [12,24] but also with cardiovascular diseases [26] and neurodegenerative disorders [27]. If these observations turn out to be valid, it is possible that MN frequency can be employed in the screening of high-risk populations [28].

## Conclusions

Our results indicate that sugar cane cutters exhibited increased MN frequencies compared to a control group, possibly due to exposure to emissions derived from sugar cane burning. Future studies are necessary to characterize the mechanism responsible for DNA damage in this group. Additionally, the demonstration of a significant degree of genotoxicity in these individuals clearly indicates the necessity of adopting modern, safe harvesting practices in the sugar cane industry.

## Abbreviations

MN: Micronuclei; PAHs: Polycyclic aromatic hydrocarbons; PM10: PM less than 10 microns in diameter; PM2.5: PM less than 2.5 microns in diameter.

## Competing interests

The authors declare that they have no competing interests.

## Authors’ contributions

HCSS conceived, developed and led the overall study, conducted the data reviews and the analysis, and prepared the manuscript. MSC and EHS participated in the data collection and the analysis. CS participated in the analysis. ALF and ALC helped design the study and data analysis. RMVR assisted with the data analysis and critically reviewed the manuscript. PHNS conceived and provided advice during the study development and helped in the manuscript preparation. All authors read and approved the final manuscript.

## Supplementary Material

Additional file 1 Micronuclei/1000 cells data. Click here for file
